# On Hypodermic Injection of Ergotine in Certain Cases of Acute Mania

**Published:** 1875-12

**Authors:** 


On Hypodermic Injection of Ergotine in Certain Cases of Acute Mania.—In the *Allgemeine Zeitschrift fur Psychiatrie,* Band xxxii. Heft 2, Dr A. H. van Andel reports the results of cases of mania in which he has injected ergotine, together with his reasons for pursuing this method of treatment. The author first gives a short description of the class of cases to which he considers this plan most applicable; they are those in which a previously healthy patient, after a week or two's restlessness, suddenly breaks out into a state of violent mania, with raised temperature and symptoms of congestion in the head, such as flushed face, injected conjunctivæ, throbbing carotids, contracted pupils. In these cases of acute delirious mania opium is contra-indicated; tartar emetic in large doses quiets the patient, but injures the digestion, and fails to cure the insanity; prolonged baths or the wet sheet are of doubtful benefit. Ergotine had previously attracted attention by its use in the treatment of migraine, and Crichton Browne had given it in some cases of epileptic insanity and chronic mania without much result. Browne-Sequard saw the vessels of the meninges contract after the injection of ergotine, and this was confirmed by Hermanides. Van Andel first used this treatment in the autumn of 1873. His patient was a female epileptic in the "status epilepticus;" she had had a similar attack two years before, which had lasted a long time, and was not perceptibly benefited by the usual methods of treatment, viz., ice to the head, leeches, bromide of potassium, morphia subcutaneously, etc. Both in this case and in another similar one, in which death from exhaustion was feared, great good seems to have resulted from the new treatment; in the former case the attack did not last one-third so long as the previous one had done. Each injection consisted of one decigramme (about one and a half grains) of ergotine in half a gramme each of glycerine and rectified spirit.

Another case is related pretty fully by the author. It is that of a robust sailor, aged forty, who, in one fortnight, had three distinct attacks of furious mania, each lasting several days. The latter two of these outbursts were treated by injections of ergotine, and the application of ice, with good results. The patient has since remained quite well. In all, fifteen injections were made in the fornight, the same dose as above being given twice daily when required. The symptoms of cerebral congestion disappeared with each remission of the symptoms. This was the only case in which the injections were followed by small local abscesses, but these gave little trouble; in some other cases small tender swellings formed at the seat of injection. No disagreeable local results have been caused in cases where ergotine has been administered in this way for *post-partum* hemorrhage.

The author has now employed this method of treatment in many cases to counteract hyperæmia of the nervous centres. He reports the usual effects to be lessening of excitement, the gradual cessation of raving, crying, and storming, the patient, although confused, becoming more manageable, and that sometimes the injection was followed by a refreshing sleep. As cases of acute delirium are often of short duration, and frequently are never sent to an asylum, it is desirable that any method of treatment which is followed by markedly beneficial results, should be known to the profession at large; but, as the author rightly remarks, further observant is needed, aided, if possible, by thermometer and sphygmograph.—*Lond. Med. Record,* Sept. 15, 1875.

